# Bisphenol A Deranges the Endocannabinoid System of Primary Sertoli Cells with an Impact on Inhibin B Production

**DOI:** 10.3390/ijms21238986

**Published:** 2020-11-26

**Authors:** Gianna Rossi, Beatrice Dufrusine, Anna Rita Lizzi, Carla Luzi, Alessandra Piccoli, Filomena Fezza, Roberto Iorio, Gabriele D’Andrea, Enrico Dainese, Sandra Cecconi, Mauro Maccarrone

**Affiliations:** 1 Department of Life, Health and Environmental Sciences, University of L’Aquila, 67100 L’Aquila, Italy; gianna.rossi@univaq.it (G.R.); sandra.cecconi@univaq.it (S.C.); 2 Faculty of Biosciences, and Technology for Food Agriculture and Environment, University of Teramo, 64100 Teramo, Italy; bdufrusine@unite.it (B.D.); edainese@unite.it (E.D.); 3 Department of Applied Clinical and Biotechnological Sciences, University of L’Aquila, 67100 L’Aquila, Italy; annarita.lizzi@univaq.it (A.R.L.); carla.luzi@univaq.it (C.L.); roberto.iorio@univaq.it (R.I.); gabriele.dandrea@cc.univaq.it (G.D.); 4 Department of Medicine, Campus Bio-Medico University of Rome, 00128 Rome, Italy; a.piccoli@unicampus.it; 5 Department of Experimental Medicine and Surgery, Tor Vergata University of Rome, 00133 Rome, Italy; filomena.fezza@uniroma2.it; 6 European Center for Brain Research, Santa Lucia Foundation IRCCS, 00142 Rome, Italy

**Keywords:** endocrine disruptor, inhibin B, male reproduction, receptor signalling, spermatogenesis

## Abstract

Bisphenol A (BPA) is an endocrine disruptor that negatively affects spermatogenesis, a process where Sertoli cells play a central role. Thus, in the present study we sought to ascertain whether BPA could modulate the endocannabinoid (eCB) system in exposed mouse primary Sertoli cells. Under our experimental conditions, BPA turned out to be cytotoxic to Sertoli cells with an half-maximal inhibitory concentration (IC_50_) of ~6.0 µM. Exposure to a non-cytotoxic dose of BPA (i.e., 0.5 μM for 48 h) increased the expression levels of specific components of the eCB system, namely: type-1 cannabinoid (CB_1_) receptor and diacylglycerol lipase-α (DAGL-α), at mRNA level, type-2 cannabinoid (CB_2_) receptor, transient receptor potential vanilloid 1 (TRPV1) receptors, and DAGL-β, at protein level. Interestingly, BPA also increased the production of inhibin B, but not that of transferrin, and blockade of either CB_2_ receptor or TRPV1 receptor further enhanced the BPA effect. Altogether, our study provides unprecedented evidence that BPA deranges the eCB system of Sertoli cells towards CB_2_- and TRPV1-dependent signal transduction, both receptors being engaged in modulating BPA effects on inhibin B production. These findings add CB_2_ and TRPV1 receptors, and hence the eCB signaling, to the other molecular targets of BPA already known in mammalian cells.

## 1. Introduction

Bisphenol A (BPA) is widely used to manufacture polycarbonate plastic and epoxy resin for the production of a multitude of consumer products, such as plastic bottles, food and drink containers, dental sealants, medical devices, and even toys and baby bottles [1]. Unsurprisingly, BPA has been detected in human colostrum [2], breast milk [3], urine [4], and saliva [5]. Unfortunately, BPA belongs to the family of endocrine disruptors (EDs), a kind of environmental chemical able to interfere with hormonal function, and hence disrupt hormone-dependent signaling with a severe impact on human health, reproductive events included [6,7,8,9]. Indeed, BPA and other EDs have been shown to negatively affect fertility, not only in females [10,11,12] but also in males [10,11,13,14,15,16].

In testis, Sertoli cells quantitatively and qualitatively support the spermatogenesis by providing a complex nutritional and regulative network indispensable for germ cell maintenance and development [17,18]. BPA specifically hits Sertoli cells by: (i) inducing their proliferation via the expression of metabolic enzymes and oxidative stress-related proteins [19]; (ii) promoting their apoptosis, and consequently that of germ cells, via different signaling pathways [20]; (iii) perturbing actin cytoskeleton [20]; and (iv) damaging tight junctions [14]. Other EDs too, such as dioxins, hamper Sertoli cells expression of secretory products and of specific proteins involved in cell–cell interaction [21]. Altogether, these studies support that the damage of Sertoli cells done by environmental pollutants contribute to the impairment of the spermatogenesis.

In recent years, lipid signals collectively termed “endocannabinoids (eCBs)” have emerged as key-regulators of diverse pathophysiologic processes, and have attracted considerable attention for their potential as diagnostic and/or therapeutic tools in human infertility. On the female side, eCBs regulate folliculogenesis, embryo oviductal transport, blastocyst development, implantation, decidualization, placentation and labor [22]. On the male side, they play a key role in controlling spermatogenesis, the acquisition of sperm fertilizing ability (i.e., sperm viability and motility), capacitation, and sperm-oocyte fusion [23]. The best-characterized eCBs are *N*-arachidonoylethanolamine, also known as anandamide (AEA), and 2-arachidonoylglycerol (2-AG). These bioactive lipids act by binding to type-1 and type-2 cannabinoid receptors (CB_1_ and CB_2_), to the G protein coupled orphan receptor 55 (GPR55), and to transient receptor potential vanilloid type 1 (TRPV1); in addition, the biological activity of eCBs is tightly controlled by metabolic enzymes that synthesize and cleave AEA (*N*-acylphosphatidylethanolamines-specific phospholipase D (NAPE-PLD), and fatty acid amide hydrolase (FAAH), respectively), or 2-AG (diacylglycerol lipases (DAGL) α and β, and monoacylglycerol lipase (MAGL), respectively) [24].

In previous studies, we have shown that Sertoli cells express the biochemical tools to metabolize AEA, in particular its cleaving enzyme FAAH and its binding CB_2_ receptor [25,26]. We also showed that AEA signaling plays a major role on Sertoli cell survival and death [17,26], with apparent consequences on the whole spermatogenesis [27,28,29].

Interestingly, BPA has been shown to derange eCB signaling in adult zebrafish and in human hepatocytes [30], much alike other EDs such as phthalates [31]. In addition, BPA has been shown to down-regulate CB_1_ expression in the hypothalamus of male mice, with subsequent anorexigenic effects [32]. Furthermore, environmentally relevant concentrations of BPA significantly impair spermatogenesis, not only in experimental animal models [33,34] but also in humans [35]. Of note, neonatal exposure to BPA inhibits the correct formation of blood testis barrier [20,36,37]. In any case, it is surprising that only a few studies aimed to identify the specific effects of BPA on Sertoli cells proliferation and functions. Thus, against this background we have been led to investigate whether in exposed mouse primary Sertoli cells BPA could alter the expression levels of specific eCB system components. Moreover, we tested two functional markers of Sertoli cells (transferrin and inhibin B) [38], with the aim of interrogating a possible impact of BPA on cell function through modulation of eCB signaling.

## 2. Results

### 2.1. Effect of BPA on Sertoli Cell Viability

**Figure 1:**
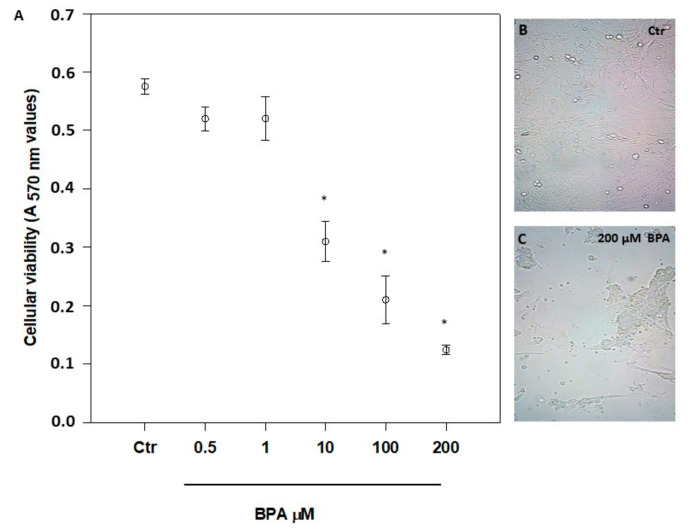
Effect of increasing concentrations of BPA on mouse Sertoli cell viability (**A**), and morphology (**B**,**C**), after 48 h of treatment. Magnification, ×200. Data are expressed as means ± SEM of 5 independent experiments. * *p* < 0.01 vs. vehicle-treated controls (Ctr).

The possible cytotoxicity of BPA was checked by exposing 7-day-old mouse Sertoli cells for 48 h to a concentration range from 1 pM to 200 μM (Figure 1A, and data not shown), as reported elsewhere [14,19,20,30,34]. A significant cytotoxicity was evident only at concentrations >1 μM (*p* < 0.01 vs. Ctr), with an overt change of morphology at 200 µM BPA (Figure 1B,C). From the viability data shown in Figure 1A, a half maximal inhibitory concentration (IC_50_) value of 6.2 ± 2.0 μM was calculated. Based on these data, all further experiments were performed at the non-cytotoxic dose of 0.5 μM, in order to avoid cell poisoning and at the same time to reveal specific effects of BPA in living cells. Incidentally, the latter BPA concentration is very close to the value previously measured in human urine (0.65 µM) [39] and, most notably, in human follicular fluid (0.66 µM) [40].

### 2.2. Effect of BPA on Gene Expression of eCB System Elements

**Figure 2:**
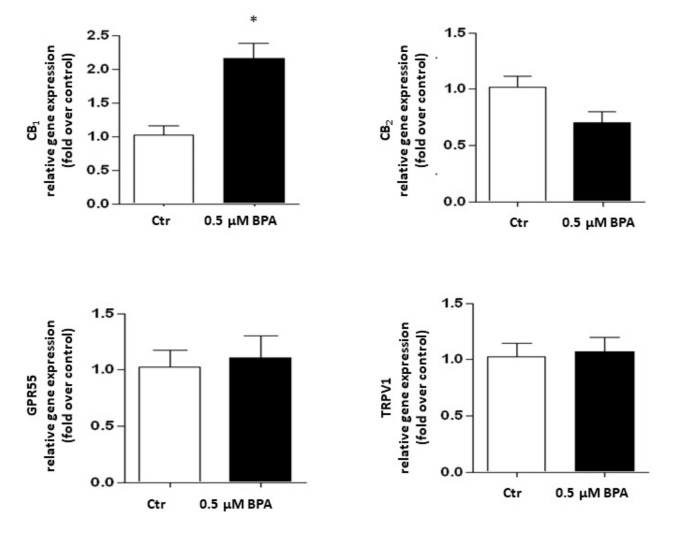
Effect of BPA (0.5 μM for 48 h) on mRNA levels of eCB-binding receptors (CB_1_, CB_2_, GPR55 and TRPV1) in exposed Sertoli cells, compared to vehicle-treated controls (Ctr). Gene expression is reported as 2^−ΔΔCt^ values calculated by ΔΔCt method vs. Ctr, posed equal to 1.0. Expression levels of each gene were normalized to β-actin, and data were expressed as means ± SEM of 5 independent experiments. * *p* < 0.01 vs. Ctr.

**Figure 3:**
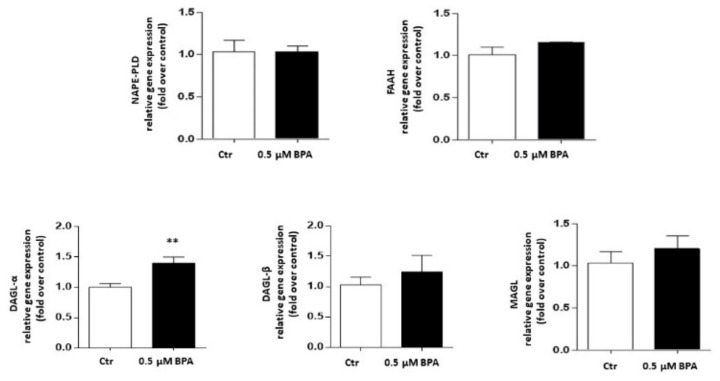
Effect of BPA (0.5 μM for 48 h) on mRNA levels of eCB metabolic enzymes (NAPE-PLD, FAAH, DAGL-α, DAGL-β and MAGL) in exposed Sertoli cells, compared to vehicle-treated controls (Ctr). Gene expression was reported as 2^−ΔΔCt^ values calculated by ΔΔCt method vs. Ctr, posed equal to 1.0. Expression levels of each gene were normalized to β-actin, and data were reported as means ± SEM of 5 independent experiments. ** *p* < 0.05 vs. Ctr.

Gene expression of the major components of the eCB system was analyzed in Sertoli cells treated with 0.5 μM BPA or with a vehicle (Control, Ctr) for 48 h. As shown in Figure 2, a significant increase of mRNA level was observed only for the CB_1_ gene (*p* < 0.01 vs. Ctr), whereas levels of genes encoding for other eCB-binding receptors, i.e., CB_2_, GPR55 and TRPV1 channel, were almost comparable to controls (*p* > 0.05 vs. Ctr). Concerning the eCB metabolic enzymes, BPA significantly increased the mRNA level of DAGL-α, which synthesizes 2-AG (*p* < 0.05 vs. Ctr), leaving nearly unchanged the levels of either NAPE-PLD, which synthesizes AEA, or DAGL-β and MAGL, which synthesizes and cleaves 2-AG, respectively (*p* > 0.05 vs. Ctr; Figure 3). In this context it should be recalled that the receptors and metabolic enzymes tested here represent the major elements of the eCB system known so far [24].

### 2.3. Effect of BPA on Protein Expression of eCB System Components

**Figure 4:**
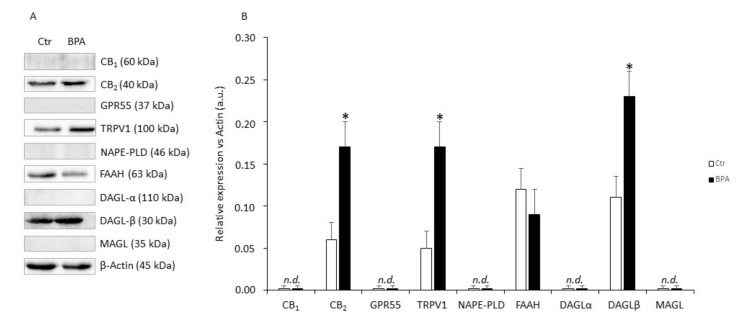
Protein expression levels of eCB system in mouse Sertoli cells. (**A**) Representative Western blot and (**B**) densitometric analysis of eCB-binding receptors and metabolic enzymes in Sertoli cells exposed to BPA (0.5 μM for 48 h) or to vehicle (Ctr). Data were expressed as mean values (arbitrary units a.u.) ± SEM (*n* = 3 independent experiments) after normalization with β-actin, used as loading control. * *p* < 0.05 vs. Ctr.

In these experiments, the effects of BPA (0.5 μM for 48 h) on protein expression of eCB system components were evaluated. As shown in Figure 4, in comparison with untreated controls Sertoli cells exposed to BPA expressed significantly higher levels of CB_2_ (+140%), TRPV1 (+250%), and DAGL-β (+130%) proteins (in all these cases: *p* < 0.05 vs. Ctr), and a trend towards reduced FAAH (−15%; *p* > 0.05 vs. Ctr). Conversely, CB_1_, GPR55, NAPE-PLD, DAGL-α and MAGL proteins in all cases were undetectable under our experimental conditions (*p* > 0.05).

### 2.4. Effect of BPA on Production of Inhibin B and Transferrin by Sertoli Cells

**Figure 5:**
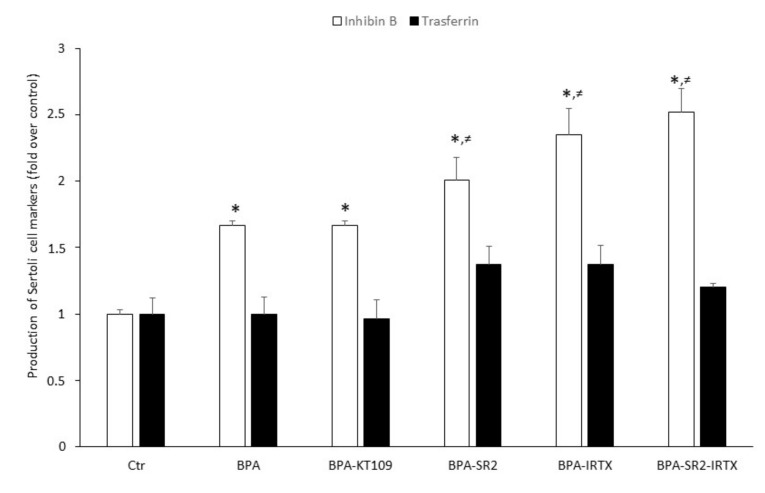
Production of inhibin B and transferrin by mouse Sertoli cells. Cells were cultured for 48 h in the presence of vehicle (Ctr), or of 0.5 μM BPA alone or with 1 μM of KT109, SR144528 (SR2), IRTX, SR2 + IRTX. Results of 3 independent experiments were expressed as fold increase over controls, posed equal to 1.0. * *p* < 0.05 vs. Ctr; ^≠^ *p* < 0.05 vs. BPA-treated cells.

To ascertain whether increased BPA-dependent expression of CB_2_, TRPV1 and DAGL-β proteins (and hence their functional activities) could modulate Sertoli cell functions, inhibin B and transferrin production were determined by means of quantitative ELISA tests with specific anti-inhibin B and anti-transferrin antibodies. To this end, Sertoli cells were exposed to 0.5 μM BPA for 48 h in the absence or in the presence of: (i) the selective DAGL-β inhibitor KT109 [41]; (ii) the selective CB_2_ antagonist SR144528 [42]; the selective TRPV1 antagonist iodoresinferatoxin [42]; or (iii) a combination of the last two compounds. BPA as such was found to significantly increase the production of inhibin B compared to vehicle-treated Sertoli cells (0.335 ± 0.040 vs.0.200 ± 0.025; *p* < 0.05). A similarly increased value was found when cells were treated with a combination of BPA and KT-109 (Figure 5). Although small, a still significantly higher increase of inhibin B production was detected when cells grown in the presence of BPA were exposed to the two CB_2_ and TRPV1 antagonists separately (SR144528: 0.412 ± 0.020 and iodoresinferatoxin: 0.469 ± 0.050, vs. 0.335 ± 0.040; *p* < 0.05) or both antagonists in combination (SR144528 + iodoresinferatoxin: 0.503 ± 0.08 vs. 0.335 ± 0.040; *p* < 0.05). Finally, transferrin production by Sertoli cells, grown in the absence or in the presence of BPA or a combination of BPA and inhibitor/antagonists, did not significantly increase compared to controls.

## 3. Discussion

Sertoli cells are targets of a variety of toxic EDs that induce reproductive dysfunctions [19,20,37]. Environmentally-relevant concentrations of BPA significantly impair spermatogenesis not only in experimental animal models [33,34] but also in humans [35]. In this scenario, mouse prepubertal Sertoli cells are commonly used to determine the impact of xenobiotics [43], because early postnatal life represents the most critical phase for the determination of the final number of Sertoli cells and spermatozoa, which establish the spermatogenic capacity of adult subjects [44,45].

Under our experimental conditions, BPA was cytotoxic at concentrations higher than 1 μM and with an IC_50_ value (6.2 ± 2.0 μM) comparable to one of the two IC_50_ values previously reported in mouse Sertoli TM4 cells [19]. However, in the latter immortalized cells BPA showed a biphasic effect [19], which was not apparent in our experiments. This feature could be due to the different experimental conditions used, and/or to a different sensitivity of the two cell types to BPA. Remarkably, in zebrafish male gonads BPA was effective at 0.04 µM [34], a dose that is 2 orders of magnitude lower than the IC_50_ value we found in mouse Sertoli cells. This finding highlights the limits of translating data from the zebrafish vertebrate model to mammals or even to humans [46]. At any rate, at the non-toxic concentrations utilized here, BPA was able to modulate gene and protein expression of specific eCB system components. Indeed, our results demonstrate that CB_1_ and DAGL-α mRNAs accumulate in exposed Sertoli cells (Figure 2 and Figure 3, respectively). However, in BPA-treated cells CB_2_, TRPV1 and DAGL-β protein contents were significantly higher than controls, and FAAH protein content was lower. Interestingly, the present findings are in keeping with our previous reports that documented CB_2_ and FAAH expression in Sertoli cells [25,26], and extend those data also to TRPV1 and DAGL-β. They also support the general notion that DAGL-α protein is predominantly expressed in the brain, whereas DAGL-β is more abundant in non-neuronal cells [47,48]. Intriguingly, similar disparities between changes in mRNA abundance and protein content have been already described in other cell systems [49], and have been previously observed also within the eCB system by others [50], and by us [51,52,53].

Overall, these data suggest that BPA enhanced eCB signaling triggered by CB_2_ and TRPV1, an effect that is possibly further supported by increased levels of 2-AG synthesized by DAGL-β. In fact, 2-AG is an agonist at both CB_2_ [54] and TRPV1 [55]. In keeping with these findings, 2-AG rather than AEA has been shown to contribute to normal progression of spermatogenesis via CB_2_ signaling [56,57], and also in zebrafish BPA has been shown to increase 2-AG levels [30].

The likely possibility that upregulation of CB_2_, TRPV1 and DAGL-β could be engaged in the effect of BPA on specific Sertoli cell functions has been confirmed by the finding that BPA significantly increases inhibin B, but not transferrin, production. Such an ability of BPA to increase inhibin B has been already described in immature rat Sertoli cells [58,59], and is shared with other environmental pollutants [60]. Interestingly, blockade of CB_2_ or TRPV1 receptors further enhanced the effect of BPA on inhibin B production, suggesting that both receptors are able to counterbalance it. Although signal transduction pathways triggered by CB_2_ and TRPV1 are largely different [42], blocking both receptors at the same time did not further potentiate BPA effect, suggesting that activation of one of them was enough to prevent BPA effect. Moreover, inhibition of DAGL-β in the presence of BPA had no effect on inhibin B production, probably due to a compensation of 2-AG biosynthesis by one of the alternative biosynthetic pathways known [24]. At any rate, the observation that eCB signaling is enhanced by BPA appears in line with the widely recognized pro-homeostatic role of eCBs [23], also in reproductive events [61,62,63,64]. From our results, CB_2_ and TRPV1 receptors can be added to the list of other known targets of BPA, such as estrogen receptors α [65] and β [66], androgen receptor [67], peroxisome proliferator-activated receptor γ and thyroid hormone receptor [68], G protein-coupled receptor 30 [69], membrane-bound estrogen receptor [70], and in Sertoli cells Fas/FasL, JNKs/p38 MAPK and NF-kB [71]. The molecular chain of events by which BPA stimulates inhibin B remains a relevant issue to be further investigated in independent studies. In this context, it seems noteworthy that previous reports have pointed to caspase 3 as an executioner of BPA in controlling mouse Sertoli cell morphology and viability [72]. Moreover, Sertoli cell adhesion molecules could be impaired upon BPA exposure [73], thus possibly contributing to its overall effect. At present, it remains to be ascertained whether engagement of caspase 3 and/or adhesion molecules might occur through eCB signaling. Certainly, a complex relationship exists among follicle-stimulating hormone (FSH), inhibin B and spermatogenesis. Inhibin B suppresses FSH secretion from the pituitary, thus contributing to the control of FSH-dependent stimulation of Sertoli cell proliferation. Indeed, circulating inhibin B can be considered as a “measure” of Sertoli cell number and sperm concentration [74]. In adult men, high levels of inhibin B are indicative of normal fertility, while low levels are associated with germ cell depletion [75]. However, when an abnormal rise of inhibin B level is accomplished by the decrease of FSH release, both markers indicate spermatogenic injury [76]. In mouse testis, a low level of inhibin B has been detected from post-natal day (PND) 1 to 6, and its expression raises from PND 18 onward [77]. Thus, since in our experiments we utilized Sertoli cells collected from 7-day-old mice, the rise of inhibin B content following BPA stimulation could be due to a deregulation of hypothalamic-pituitary-gonadal axis or of inhibin B expression. Since we found that CB_2_ and TRPV1 antagonism further increases inhibin B production, this might indicate a possible deregulation of FSH signaling by BPA, especially because of FSH-dependent modulation of eCB signaling in immature Sertoli cells [17,78]. Nevertheless, since inhibin B release is regulated not only by FSH but also by testosterone and even germ cells, the existence of multiple pathways acting synergistically to influence Sertoli cell differentiation makes the identification of a dysregulative mechanism more complex. Finally, the finding that transferrin level is not altered neither by BPA nor by BPA plus eCB system-targeting compounds is not surprising. Indeed, a constitutive secretion of transferrin occurs in primary Sertoli cell cultures from immature mice [79], and the lack of FSH in our culture conditions rules out well-known gonadotropin-dependent stimulative effects [80].

## 4. Materials and Methods

### 4.1. Chemicals

Chemicals were of the purest analytical grade. MEM, glutamine, PES, DMSO, collagenase, DNase1, trypsin, BPA (Bisphenol A), ABTS (2,2′-azinobis [3-ethylbenzothiazoline-6-sulfonic acid]-diammonium salt), and hyaluronidase were purchased from Sigma (St. Louis, MO, USA). Rabbit anti-CB_1_, anti-CB_2_, anti-GPR55, anti-FAAH, anti-DAGL-α, anti-DAGL-β, anti-MAGL, anti-NAPE-PLD polyclonal antibodies, and KT109 were purchased from Cayman Chemicals (Ann Arbor, MI, USA). Mouse anti-actin antibody was obtained from Santa Cruz Biotechnology, Inc. (Santa Cruz, CA, USA), and rabbit anti-TRPV1 polyclonal antibody was from OriGene Technologies (Rockville, MD, USA). Rabbit anti-inhibin B polyclonal antibody was obtained from Elabscience Biotechnology (Houston, TX, USA), and rabbit anti-transferrin polyclonal antibody was from Proteintech Group (Chicago, IL, USA). Iodoresinferatoxin was obtained from Tocris Cookson (Bristol, UK), whereas SR144528 was a kind gift from Sanofi-Synthelabo (Montpellier, France).

### 4.2. Animals

Mouse *(Mus musculus*) Swiss CD1 males (7-day-old; Charles River Laboratories, Lecco, Italy) were housed in an animal facility under controlled temperature (21 ± 1 °C) and light (12 h light/day) conditions. A total number of 60 mice were used to perform the study.

### 4.3. Ethical Approval

All experimental procedures involving animals and their care were performed in conformity with national and international laws and policies (European Economic Community Council Directive 86/609,OJ 358, 1, 12 December, 1987; European Parliament Council Directive 2010/63/EU, OJ L 276, 20 October 2010; Italian Legislative Decree 116/92, Gazzetta Ufficiale della Repubblica Italiana n. 40, 18, February 1992; National Institutes of Health Guide for the Care and Use of Laboratory Animals, NIH publication no. 85–23, 1985). The method of euthanasia consisted of an inhalant overdose of carbon dioxide (CO_2_, 10–30%), followed by cervical dislocation. All efforts were made to minimize animal suffering.

### 4.4. Sertoli Cell Culture

Purified Sertoli cells were isolated from decapsulated testes of 7-day old CD1 mice, as previously described [25]. Sertoli cells (95% pure) were cultured at 34 °C and 5% CO_2_ for 48 h in serum and phenol-free Dulbecco’s Modified Eagle’s Medium-high glucose, as previously reported [25], supplemented with 2 mM glutamine and penicillin-streptomycin solution, in the presence of DMSO (0.1%) vehicle for controls (Ctr), or with the indicated concentrations of BPA, alone or in the presence of receptor antagonists (CB_2_ antagonist: 1 μM SR144528 [81]; TRPV1 antagonist: 1 μM iodoresinferatoxin [82,83]) or DAGL-β inhibitor: 1 μM KT109 [84].

### 4.5. Cytotoxicity Assay

The number of viable cells was evaluated by a colorimetric MTT (3-(4,5-dimethylthiazol-2-yl)-2,5-diphenyltetrazolium bromide) assay that assesses metabolic integrity [85]. Briefly, Sertoli cells were seeded in 96-well plates, and were treated with DMSO (0.1%) vehicle (Ctr), or with increasing concentrations of BPA (from 1 pM to 200 μM). After 48 h of treatment, 10 μL of MTT solution was added to each well at a final concentration of 0.5 mg/mL, and plates were incubated at 37 °C for 2 h. The amount of MTT-formazan product, dissolved in acidified isopropanol, was estimated by measuring the absorbance at 570 nm in a microplate reader (Model 550, BioRad, Hercules, CA, USA).

### 4.6. Analysis of Gene Expression

Messenger RNAs were extracted from control or BPA-exposed Sertoli cells using TRIzol Plus RNA Purification Kit (Invitrogen, Carlsbad, CA, USA), as per manufacturer’s instructions, and were quantitated spectrophotometrically (NanoDrop, Thermo Scientific ND-2000c). One μg of total mRNA was reverse-transcribed to cDNA, using SuperScript Vilo™ Master Mix (Invitrogen, Carlsbad, CA, USA). Quantitative PCR analysis was performed using SYBR Green I Master and the LightCycler 480 System (Roche, Basel, Switzerland) on a DNA Engine Opticon 2 Continuous Fluorescence Detection System (BioRad, Hercules, CA, USA). The reaction was performed using the following qRT-PCR program: 95 °C for 10 min, followed by 40 amplification cycles of 95 °C for 10 s, optimal annealing temperature (see Table 1) for 30 s, and 72 °C for 30 s. The primers used for the amplification are listed in Table 1, with pertinent references. All data were normalized to the endogenous reference gene β-actin. Relative quantitation of mRNAs was performed by the comparative ΔΔ*C*t method [22,86].

### 4.7. Western Blotting

At the end of 48 h culture, control or BPA-exposed Sertoli cells were resuspended in RIPA buffer containing a protease inhibitors cocktail for 30 min. The cell lysates were centrifugated for 15 min at 12,000× *g* at 4 °C, and the resulting supernatants were collected. Lysates (40 μg/sample) were separated on a 12.5% SDS-page and transferred to polyvinylidene difluoride (PVDF) (Hybond C Extra, Amersham, Little Chalfont, UK). Membranes were blocked with 5% (w/w) nonfat dry milk and incubated at 4 °C with specific primary antibodies: anti-actin (1:200); anti-CB_1_ (1:200); anti-CB_2_ (1:200); anti-GPR55 (1:200); anti-TRPV1 (1:1000); anti-MAGL (1:200); anti-FAAH (1:200); anti-DAGL-α (1:1000); anti-DAGL-β (1:1000); anti-NAPE-PLD (1:200); anti-inhibin B (1:400), anti-transferrin (1:200), and then for 1 h with a peroxidase-conjugated anti-mouse (1:20,000) or anti-rabbit (1:10,000) secondary antibody. After detection with a chemiluminescence reagent (ECL, Pierce, Rockfort, IL, USA), densitometric quantification was performed with the public domain software NIH Image v.1.62, and was standardized to β-actin (1:200) as a loading control.

### 4.8. ELISA Assay

Sertoli cells were cultured for 48 h, in the presence of vehicle (Ctr) or of BPA, alone or supplemented with 1 μM of: (a) KT-109; (b) SR144528 (SR2); (c) IRTX; or (d) the combination IRTX + SR2. Protein expression of inhibin B and transferrin was determined by enzyme linked immunosorbent assay (ELISA), as reported [89]. Briefly, wells were coated with Sertoli cell lysates (20 µg/well) in coating buffer (0.05 M Na_2_CO_3_, pH 9.6) for 2 h at room temperature. Then, they were incubated with 1% bovine serum albumin (BSA) in phosphate-buffered saline (PBS) for 1 h, and then for 2 h with anti-transferrin or anti-inhibin B polyclonal antibodies diluted to 1:500 and 1:1000, respectively, with 1% BSA in PBS. In preliminary experiments, these antibodies were found to recognize a single immunoreactive band in Sertoli cell extracts, of the molecular weight expected for inhibin B (45 kDa) and transferrin (77 kDa), respectively. After rinsing three times with 1% BSA in PBS-Tween 20, 100 µl horseradish peroxidase (HRP)-conjugated secondary antibody (diluted 1:5000) were added, and the ELISA plate was further incubated for 1 h at room temperature. Afterwards, enzymatic activity of HRP was determined by adding 100 µl/well of ABTS, then stopped with 100 µl of stop solution (1% SDS), and finally absorbance at 405 nm was measured in a Multiskan ELISA Microplate Reader (ThermoLabsystems, Bevery, MA, USA). The linearity ranges of ELISA against inhibin B and transferrin were ascertained by dose-dependence curves with different amounts (0–40 µg/well) of Sertoli cell extracts. All data were within these linearity ranges, and were expressed as fold increase over controls, posed equal to 1.0.

### 4.9. Statistical Analysis

Unless otherwise indicated, data are shown as mean ± SEM of at least three different experiments. The Prism 6 programme of GraphPad software (San Diego, CA, USA) was used to test the statistical significance of differences between group means. Comparisons between multiple groups were performed by ANOVA test, followed by Dunnett’s and Duncan’s test. Half-maximal inhibitory concentration (IC_50_) values were calculated using the OriginPro software version 8.5 (OriginLab Corporation, Northampton, Massachusetts). Changes in gene and protein expression were analyzed using unpaired *t*-test. Level of *p* < 0.05 was regarded as statistically significant.

## 5. Conclusions

Our present results provide unprecedented evidence that the endocrine disruptor BPA deranges the eCB system of primary mouse Sertoli cells towards CB_2_- and TRPV1-dependent signaling. They also show that both eCB-binding receptors contribute to the effect of BPA on inhibin B production, suggesting their engagement in the modulation of Sertoli cell function. Even though it seems premature to draw any conclusion on the molecular clues at the basis of the overall effect of BPA on Sertoli cells [90], our data add CB_2_ and TRPV1 to the already known molecular targets of BPA in mammalian cells.

## Figures and Tables

**Table 1:** List of the primers used for qRT-PCR analysis.

Gene	Corresponding Protein	PCR Primers	Annealing T (°C)	Reference
*Cnr1*	CB_1_	Fw: 5′-CCAAGAAAAGATGACGGCAG-3′ Rev: 5′-AGGATGACACATAGCACCAG-3′	57	[87]
*Cnr2*	CB_2_	Fw: 5′-TCGCTTACATCCTTCAGACAG-3′ Rev: 5′-TCTTCCCTCCCAACTCCTTC-3′	57	[87]
*Gpr55*	GPR55	Fw: 5′-ATTCGATTCCGTGGATAAGC-3′ Rev: 5′- ATGCTGATGAAGTAGAGGC -3′	57	[88]
*Trpv1*	TRPV1	Fw: 5′-TGAACTGGACTACCTGGAAC-3′ Rev: 5′-TCCTTGAAGACCTCAGCATC-3′	57	[88]
*Magl*	MAGL	Fw: 5′-TTGTAGATACTGGAAGCCC-3′ Rev: 5′-ATGGTGTCCACGTGTTGCAGC-3′	62	[52]
*Faah*	FAAH	Fw: 5′- AGATTGAGATGTATCGCCAG-3′ Rev: 5′- CTTCAGAATGTTGTCCCAC-3′	56	[78]
*Dagl-α*	DAGL-α	Fw: 5′- AATGGCTATCATCTGGCTGAGC-3′ Rev: 5′-TTCCGAGGGTGACATTCTTAGC-3′	60	[52]
*Dagl-β*	DAGL-β	Fw: 5′- TGTCAGCATGAGAGGAACCAT-3′ Rev: 5- CGCCAGGCGGATATAGAGC-3′	60	Designed by Primer blast software
*Nape-pld*	NAPE-PLD	Fw: 5′-AAGTGTGTCTTCTAGGTTCTCC-3′ Rev: 5′-TTGTCAAGTTCCTCTTTGGAACC-3′	62	[52]
*Act-β*	β-actin	Fw: 5′-TGTTACCAACTGGGACGA-3′ Rev: 5′-GTCTCAAACATGATCTGGGTC-3′	56	[78]

## Abbreviations

ABTS	2:2′-Azinobis [3-ethylbenzothiazoline-6-sulfonic acid]-diammonium salt
2-AG	2-Arachidonoylglycerol
AEA	*N*-Arachidonoylethanolamine
AU	Arbitrary units
BPA	Bisphenol A
BSA	Bovine serum albumin
CB_1_	Type-1 cannabinoid receptor
CB_2_	Type-2 cannabinoid receptor
Ctr	Control
DAGL-α	Diacylglycerol lipase-α
DAGL-β	Diacylglycerol lipase-β
DMSO	Dimethyl sulfoxide
eCBs	Endocannabinoids
ED	Endocrine disruptors
ELISA	Enzyme-linked immunosorbent assay
FAAH	Fatty acid amide hydrolase
GPR55	G protein-coupled receptor 55
HRP	Horseradish peroxidase
IRTX	Iodoresinferatoxin
MAGL	Monoacylglycerol lipase
MTT	3-(4,5-dimethylthiazol-2-yl)-2,5-diphenyltetrazolium bromide)
NAPE-PLD	*N*-acyl phosphatidylethanolamine-specific phospholipase D
PBS	Phosphate-buffered saline
SR144528 (SR2)	5-(4-Chloro-3-methylphenyl)-1-(4-methylbenzyl)-N-((1S,2S,4R)-1,3,3-trimethylbicyclo [2.2.1]heptan-2-yl)-1H-pyrazole-3-carboxamide
TMB	Tetramethylbenzidine
TRPV1	Transient receptor potential vanilloid type 1 channel

## References

[1] Staples C.A., Dome P.B., Klecka G.M., Oblock S.T., Harris L.R. (1998). A review of the environmental fate, effects, and exposures of bisphenol A. Chemosphere.

[2] Kuruto-Niwa R., Tateoka Y., Usuki Y., Nozawa R. (2007). Measurement of bisphenol A concentrations in human colostrum. Chemosphere.

[3] Vandenberg L.N., Chahoud I., Heindel J.J., Padmanabhan V., Paumgartten F.J.R., Schoenfelder G. (2010). Urinary, circulating, and tissue biomonitoring studies indicate widespread exposure to bisphenol A. Environ. Health Perspect..

[4] Calafat A.M., Weuve J., Ye X., Jia L.T., Hu H., Ringer S., Hutter K., Hauser R. (2009). Exposure to bisphenol A and other phenols in neonatal intensive care unit premature infants. Environ. Health Perspect..

[5] Giesbrecht G.F., Ejaredar M., Liu J., Thomas J., Letourneau N., Campbell T., Martin J.W., Dewey D., APrON Study Team (2017). Prenatal bisphenol A exposure and dysregulation of infant hypothalamic-pituitary-adrenal axis function: Findings from the APrON cohort study. Environ. Health.

[6] Colborn T., Vom Saal F., Soto A. (1993). Developmental effects of endocrine-disrupting chemicals in wildlife and humans. Environ. Health Perspect..

[7] Rochester J.R. (2013). Bisphenol A and human health: A review of the literature. Reprod. Toxicol..

[8] Kabir E.R., Rahman M.S., Rahman I. (2015). A review on endocrine disruptors and their possible impacts on human health. Environ. Toxicol. Pharmacol..

[9] Rivollier F., Krebs M.O., Kebir O. (2019). Perinatal exposure to environmental endocrine disruptors in the emergence of neurodevelopmental psychiatric diseases: Asystematic review. Int. J. Environ. Res. Public Health.

[10] Chianese R., Troisi J., Richards S., Scafuro M., Fasano S., Guida M., Pierantoni R., Meccariello R. (2018). Bisphenol A in reproduction: Epigenetic effects. Curr. Med. Chem..

[11] Brehm E., Flaws J.A. (2019). Transgenerational effects of endocrine-disrupting chemicals on male and female reproduction. Endocrinology.

[12] Rattan S., Flaws J.A. (2019). The epigenetic impacts of endocrine disruptors on female reproduction across generations. Biol. Reprod..

[13] Hong J., Chen F., Wang X., Bai Y., Zhou R., Li Y., Chen L. (2016). Exposure of preimplantation embryos to low-dose bisphenol A impairs testes development and suppresses histone acetylation of StAR promoter to reduce production of testosterone in mice. Mol. Cell. Endocrinol..

[14] Tian J., Ding Y., She R., Ma L., Du F., Xia K., Chen L. (2017). Histologic study of testis injury after bisphenol A exposure in mice. Toxicol. Ind. Health.

[15] Lymperi S., Giwercman A. (2018). Endocrine disruptors and testicular function. Metabolism.

[16] Castellini C., Totaro M., Parisi A., Settimio D’Andrea S., Lucente L., Cordeschi G., Francavilla S., Francavilla F., Barbonetti A. (2020). Bisphenol A and male fertility: Myths and realities. Front. Endocrinol..

[17] Grimaldi P., Rossi G., Catanzaro G., Maccarrone M. (2009). Modulation of the endocannabinoid-degrading enzyme fatty acid amide hydrolase by follicle-stimulating hormone. Vitam. Horm..

[18] Kopera I.A., Bilinska B., Cheng C.Y., Mruk D.D. (2010). Sertoli–germ cell junctions in the testis: A review of recent data. Philos. Trans. R. Soc. Lond. B Biol. Sci..

[19] Ge L.C., Chen Z.J., Liu H., Zhang K.S., Su Q., Ma X.Y., Huang H.B., Zhao Z.D., Wang Y.Y., Giesy J.P. (2014). Signaling related with biphasic effects of bisphenol A (BPA) on Sertoli cell proliferation: Acomparative proteomic analysis. Biochim. Biophys. Acta.

[20] Gao Y., Mruk D.D., Cheng C.Y. (2015). Sertoli cells are the target of environmental toxicants in the testis—A mechanistic and therapeutic insight. Expert Opin. Ther. Targets.

[21] Lai K.P., Wong M.H., Wong K.C. (2005). Effects of TCDD in modulating the expression of Sertoli cell secretory products and markers for cell–cell interaction. Toxicology.

[22] Cecconi S., Rapino C., Di Nisio V., Rossi G., Maccarrone M. (2020). The (endo) cannabinoid signaling in female reproduction: What are the latest advances?. Prog. Lipid Res..

[23] Maccarrone M., Bab I., Bíró T., Cabral G.A., Dey S.K., Di Marzo V., Konje J.C., Kunos G., Mechoulam R., Pacher P. (2015). Endocannabinoid signaling at the periphery: 50 years after THC. Trends Pharmacol. Sci..

[24] Maccarrone M. (2020). Missing pieces to the endocannabinoid puzzle. Trends Mol. Med..

[25] Maccarrone M., Cecconi S., Rossi G., Battista N., Pauselli R., Finazzi-Agrò A. (2003). Anandamide activity and degradation are regulated by early postnatal aging and follicle-stimulating hormone in mouse Sertoli cells. Endocrinology.

[26] Rossi G., Gasperi V., Paro R., Barsacchi D., Cecconi S., Maccarrone M. (2007). Follicle-stimulating hormone activates fatty acid amide hydrolase by protein kinase A and aromatase-dependent pathways in mouse primary Sertoli cells. Endocrinology.

[27] Grimaldi P., Di Giacomo D., Geremia R. (2013). The endocannabinoid system and spermatogenesis. Front. Endocrinol. (Lausanne).

[28] Carvalho R.K., Santos M.L., Souza M.R., Rocha T.L., Guimarães F.S., Anselmo-Franci J.A., Mazaro-Costa R. (2018). Chronic exposure to cannabidiol induces reproductive toxicity in male Swiss mice. J. Appl. Toxicol..

[29] Nielsen J.E., Rolland A.D., Rajpert-De Meyts E., Janfelt C., Jørgensen A., Winge S.B., Kristensen D.M., Juul A., Chalmel F., Jégou B. (2019). Characterisation and localisation of the endocannabinoid system components in the adult human testis. Sci. Rep..

[30] Martella A., Silvestri C., Maradonna F., Gioacchini G., Allarà M., Radaelli G., Overby D.R., Di Marzo V., Carnevali O. (2016). Bisphenol A induces fatty liver by an endocannabinoid-mediated positive feedback loop. Endocrinology.

[31] Forner-Piquer I., Maradonna F., Gioacchini G., Santangeli S., Allarà M., Piscitelli F., Habibi H.R., Di Marzo V., Carnevali O. (2017). Dose-specific effects of di-isononyl phthalate on the endocannabinoid system and on liver of female Zebrafish. Endocrinology.

[32] Suglia A., Chianese R., Migliaccio M., Ambrosino C., Fasano S., Pierantoni R., Cobellis G., Chioccarelli T. (2016). Bisphenol A induces hypothalamic down-regulation of the cannabinoid receptor 1 and anorexigenic effects in male mice. Pharmacol. Res..

[33] Cabaton N.J., Wadia P.R., Rubin B.S., Zalko D., Schaeberle C.M., Askenase M.H., Gadbois J.L., Tharp A.P., Whitt G.S., Sonnenschein C. (2011). Perinatal exposure to environmentally relevant levels of bisphenol A decreases fertility and fecundity in CD-1 mice. Environ. Health Perspect..

[34] Forner-Piquer I., Beato S., Piscitelli F., Santangeli S., Di Marzo V., Habibi H.R., Maradonna F., Carnevali O. (2020). Effects of BPA on zebrafish gonads: Focus on the endocannabinoid system. Environ. Pollut..

[35] Meeker J.D., Ehrlich S., Toth T.L., Wright D.L., Calafat A.M., Trisini A.T., Ye X.Y., Hauser R. (2010). Semen quality and sperm DNA damage in relation to urinary bisphenol A among men from an infertility clinic. Reprod. Toxicol..

[36] Cheng C.Y., Wong E.W.P., Lie P.P.Y., Li M.W.M., Su L., Siu E.R., Yan H.H.N., Mannu J., Mathur P.P., Bonanomi M. (2011). Environmental toxicants and male reproductive function. Spermatogenesis.

[37] Johnson K.J. (2015). Testicular histopathology associated with disruption of the Sertoli cell cytoskeleton. Spermatogenesis.

[38] Bhattacharya I., Basu S., Pradhan B.S., Sarkar H., Nagarajan P., Majumdar S.S. (2019). Testosterone augments FSH signaling by upregulating the expression and activity of FSH-receptor in pubertal primate Sertoli cells. Mol. Cell. Endocrinol..

[39] Calafat A.M., Ye X., Wong L.Y., Reidy J.A., Needham L.L. (2008). Exposure of the U.S. population to bisphenol A and 4-tertiary-octylphenol: 2003–2004. Environ. Health Perspect..

[40] Poormoosavi S.M., Behmanesh M.A., Janati S., Najafzadehvarzi H. (2019). Level of Bisphenol A in follicular fluid and serum and oocyte morphology in patients undergoing IVF treatment. J. Fam. Reprod. Health.

[41] Wilkerson J.L., Ghosh S., Bagdas D., Mason B.L., Crowe M.S., Hsu K.L., Wise L.E., Kinsey S.G., Damaj M.I., Cravatt B.F. (2016). Diacylglycerol lipase β inhibition reverses nociceptive behaviour in mouse models of inflammatory and neuropathic pain. Br. J. Pharmacol..

[42] Pertwee R.G., Howlett A.C., Abood M.E., Alexander S.P., Di Marzo V., Elphick M.R., Greasley P.J., Hansen H.S., Kunos G., Mackie K. (2010). International Union of Basic and Clinical Pharmacology. LXXIX. Cannabinoid receptors and their ligands: Beyond CB₁ and CB₂. Pharmacol. Rev..

[43] Moody S., Goh H., Bielanowicz A., Rippon A.P., Loveland K.L., Itman C. (2013). Prepubertal mouse testis growth and maturation and androgen production are acutely sensitive to di-*n*butyl phthalate. Endocrinology.

[44] Griswold M.D. (1998). The central role of Sertoli cells in spermatogenesis. Semin. Cell Dev. Biol..

[45] Meroni S.B., Galardo M.N., Rindone G., Gorga A., Riera M.F., Cigorraga S.B. (2019). Molecular mechanisms and signaling pathways involved in Sertoli cell proliferation. Front. Endocrinol. (Lausanne).

[46] Cho S.J., Park E., Baker A., Reid A.Y. (2020). Age bias in Zebrafish models of epilepsy: What can we learn from old fish?. Front. Cell Dev. Biol..

[47] Bisogno T., Howell F., Williams G., Minassi A., Cascio M.G., Ligresti A. (2003). Cloning of the first sn1-DAG lipases points to the spatial and temporal regulation of endocannabinoid signaling in the brain. J. Cell Biol..

[48] Hsu K.-L., Tsuboi K., Adibekian A., Pugh H., Masuda K., Cravatt B.F. (2012). DAGLβ inhibition perturbs a lipid network involved in macrophage inflammatory responses. Nat. Chem. Biol..

[49] Janitz M. (2007). Assigning functions to genes-the main challenge of the post genomics era. Rev. Physiol Biochem. Pharmacol..

[50] Colombo G., Rusconi F., Rubino T., Cattaneo A., Martegani E., Parolaro D., Bachi A., Zippel R. (2009). Transcriptomic and proteomic analyses of mouse cerebellum reveals alterations in Ras GRF1 expression following in vivo chronic treatment with delta 9-tetra-hydrocannabinol. J. Mol. Neurosci..

[51] Pasquariello N., Catanzaro G., Marzano V., Amadio D., Barcaroli D., Oddi S., Federici G., Urbani A., Finazzi Agrò A., Maccarrone M. (2009). Characterization of the endocannabinoid system in human neuronal cells and proteomic analysis of anandamide-induced apoptosis. J. Biol. Chem..

[52] Bari M., Tedesco M., Battista N., Pasquariello N., Pucci M., Gasperi V., Scaldaferri M.L., Farini D., De Felici M., Maccarrone M. (2011). Characterization of the endocannabinoid system in mouse embryonic stem cells. Stem. Cells Dev..

[53] Cecconi S., Rossi G., Oddi S., Di Nisio V., Maccarrone M. (2019). Role of major endocannabinoid-binding receptors during mouse oocyte maturation. Int. J. Mol. Sci..

[54] Sugiura T., Kishimoto S., Oka S., Gokoh M. (2006). Biochemistry, pharmacology and physiology of 2-arachidonoylglycerol, an endogenous cannabinoid receptor ligand. Prog. Lipid Res..

[55] Zygmunt P.M., Ermund A., Movahed P., Andersson D.A., Simonsen C., Jönsson B.A.G., Blomgren A., Birnir B., Bevan S., Eschalier A. (2013). Monoacylglycerols activate TRPV1—A link between phospholipase C and TRPV1. PLoS ONE.

[56] Grimaldi P., Orlando P., Di Siena S., Lolicato F., Petrosino S., Bisogno T., Geremia R., De Petrocellis L., Di Marzo V. (2009). The endocannabinoid system and pivotal role of the CB2 receptor in mouse spermatogenesis. Proc. Natl. Acad. Sci. USA.

[57] Di Giacomo D., De Domenico E., Sette C., Geremia R., Grimaldi P. (2016). Type 2 cannabinoid receptor contributes to the physiological regulation of spermatogenesis. FASEB J..

[58] Depuydt C.E., Mahmoud A.M., Dhooge W.S., Schoonjans F.A., Comhaire F.H. (1999). Hormonal regulation of inhibin B secretion by immature rat Sertoli cells in vitro: Possible use as a bioassay for estrogen detection. J. Androl..

[59] Monsees T.K., Franz M., Gebhardt S., Winterstein U., Schill W.B., Hayatpour J. (2000). Sertoli cells as a target for reproductive hazards. Andrologia.

[60] Reis M.M.S., Moreira A.C., Sousa M., Mathur P.P., Oliveira P.F., Alves M.G. (2015). Sertoli cell as a model in male reproductive toxicology: Advantages and disadvantages. J. Appl. Toxicol..

[61] Cobellis G., Meccariello R., Chianese R., Chioccarelli T., Fasano S., Pierantoni R. (2016). Effects of neuroendocrine CB1 activity on adult Leydig cells. Front. Endocrinol. (Lausanne).

[62] Barchi M., Innocenzi E., Giannattasio T., Dolci S., Rossi P., Grimaldi P. (2019). Cannabinoid receptors signaling in the development, epigenetics, and tumours of male germ cells. Int. J. Mol. Sci..

[63] Carvalho R.K., Andersen M.L., Mazaro-Costa R. (2020). The effects of cannabidiol on male reproductive system: A literature review. J. Appl. Toxicol..

[64] Cecconi S., Rossi G., Castellucci A., D’Andrea G., Maccarrone M. (2014). Endocannabinoid signaling in mammalian ovary. Eur. J. Obstet. Gynecol. Reprod. Biol..

[65] Gould J.C., Leonard L.S., Maness S.C., Wagner B.L., Conner K., Zacharewski T., Safe S., McDonnell D.P., Gaido K.W. (1998). Bisphenol A interacts with the estrogen receptor α in a distinct manner from estradiol. Mol. Cell. Endocrinol..

[66] Kuiper G.G., Lemmen J.G., Carlsson B., Corton J.C., Safe S.H., van der Saag P.T., van der Burg B., Gustafsson J.A. (1998). Interaction of estrogenic chemicals and phytoestrogens with estrogen receptor β. Endocrinology.

[67] Lee H.J., Chattopadhyay S., Gong E.Y., Ahn R.S., Lee K. (2003). Antiandrogenic effects of bisphenol A and nonylphenol on the function of androgen receptor. Toxicol. Sci..

[68] Richter A.C., Birnbaum L.S., Farabollini F., Newbold R.R., Rubin B.S., Talsness C.E., Vandenbergh J.G., Walser-Kuntz D.R., vom Saal F.S. (2007). In vivo effects of bisphenol A in laboratory rodent studies. Reprod. Toxicol..

[69] Dong S., Terasaka S., Kiyama R. (2011). Bisphenol A induces a rapid activation of Erk1/2 through GPR30 in human breast cancer cells. Environ. Pollut..

[70] Viñas R., Jeng W.J., Watson C.S. (2012). Non-genomic effects of xenoestrogen mixtures. Int. J. Environ. Res. Public Health.

[71] Qi S., Fu W., Wang C., Liu C., Quan C., Kourouma A., Yan M., Yu T., Duan P., Yang K. (2014). BPA-induced apoptosis of rat Sertoli cells through Fas/FasL and JNKs/p38 MAPK pathways. Reprod. Toxicol..

[72] Iida H., Maehara K., Doiguchi M., Mōri T., Yamada F. (2003). Bisphenol A-induced apoptosis of cultured rat Sertoli cells. Reprod. Toxicol..

[73] Menegazzo M., Zuccarello D., Luca G., Ferlin A., Calvitti M., Mancuso F., Calafiore R., Foresta C. (2011). Improvements in human sperm quality by long-term in vitro co-culture with isolated porcine Sertoli cells. Hum. Reprod..

[74] Luisi S., Florio P., Reis F.M., Petraglia F. (2005). Inhibins in female and male reproductive physiology: Role in gametogenesis, conception, implantation and early pregnancy. Hum. Reprod. Update.

[75] Stewart J., Turner K.J. (2005). Inhibin B as a potential biomarker of testicular toxicity. Cancer Biomark..

[76] Meachem S.J., Nieschlag E., Simoni M. (2001). Inhibin B in male reproduction: Pathophysiology and clinical relevance. Eur. J. Endocrinol..

[77] Kim Y., Kim J.S., Song M.S., Seo H.S., Kim J.C., Bae C.S., Kim S., Shin T., Kim S.H., Moon C. (2008). The expression and localization of inhibin isotypes in mouse testis during postnatal development. J. Vet. Sci..

[78] Grimaldi P., Pucci M., Di Siena S., Di Giacomo D., Pirazzi V., Geremia R., Maccarrone M. (2012). The faah gene is the first direct target of estrogen in the testis: Role of histone demethylase LSD1. Cell Mol. Life Sci..

[79] Huleihel M., Zeyse D., Lunenfeld E., Zeyse M., Mazor M. (2002). Induction of transferrin secretion in murine Sertoli cells by FSH and IL-1: The possibility of different mechanism(s) of regulation. Am. J. Rep. Immun..

[80] Sylvester F.R., Griswold M.D., David K. (2012). Molecular biology of iron transport in the testis. Molecular Biology of the Male Reproductive System.

[81] Dhopeshwarkar A., Mackie K. (2016). Functional selectivity of CB_2_ cannabinoid receptor ligands at a canonical and noncanonical pathway. J. Pharmacol. Exp. Ther..

[82] Bashashati M., Fichna J., Piscitelli F., Capasso R., Izzo A.A., Sibaev A., Timmermans J.P., Cenac N., Vergnolle N., Di Marzo V. (2017). Targeting fatty acid amide hydrolase and transient receptor potential vanilloid-1 simultaneously to modulate colonic motility and visceral sensation in the mouse: A pharmacological intervention with N-arachidonoyl-serotonin (AA-5-HT). Neurogastroenterol. Motil..

[83] Zhang M., Liu Y., Hu Z., Zhou Y., Pi Y., Guo L., Wang X., Chen X., Li G., Zhang L. (2017). TRPV1 attenuates intracranial arteriole remodeling through inhibiting VSMC phenotypic modulation in hypertension. Histochem. Cell Biol..

[84] Luk J., Lu Y., Ackermann A., Peng X., Bogdan D., Puopolo M., Komatsu D.E., Tong S., Ojima I., Rebecchi M.J. (2018). Contribution of diacylglycerol lipase β to pain after surgery. J. Pain Res..

[85] Mosmann T. (1983). Rapid colorimetric assay for cellular growth and survival: Application to proliferation and cytotoxicity assays. J. Immunol. Meth..

[86] Pucci M., Pasquariello N., Battista N., Di Tommaso M., Rapino C., Fezza F., Maccarrone M. (2012). Endocannabinoids stimulate human melanogenesis via type-1 cannabinoid receptor. J. Biol. Chem..

[87] Compagnucci C., Di Siena S., Bustamante M.B., Di Giacomo D., Di Tommaso M., Maccarrone M., Sette C. (2013). Type-1 (CB1) cannabinoid receptor promotes neuronal di_erentiation and maturation of neural stem cells. PLoS ONE.

[88] Pucci M., D’Addario C. (2016). Assessing gene expression of the endocannabinoid system. Methods Mol. Biol..

[89] Gasperi V., Fezza F., Pasquariello N., Bari M., Oddi S., Agrò A.F., Maccarrone M. (2007). Endocannabinoids in adipocytes during differentiation and their role in glucose uptake. Cell. Mol. Life Sci..

[90] Maccarrone M., Rapino C., Francavilla F., Barbonetti A. (2020). Cannabinoid signalling and effects of cannabis on the male reproductive system. Nat. Rev. Urol..

